# MicroRNA-29a-3p prevents *Schistosoma japonicum*-induced liver fibrosis by targeting Roundabout homolog 1 in hepatic stellate cells

**DOI:** 10.1186/s13071-023-05791-4

**Published:** 2023-06-06

**Authors:** Hongyan Kong, Qiqin Song, Wenjiang Hu, Shusen Guo, Dandan Xiang, Shuaiwen Huang, Xin Xu, Jinan He, Lanyue Pan, Ran Tao, Haijing Yu, Jiaquan Huang

**Affiliations:** 1 Department and Institute of Infectious Disease, Tongji Hospital, Tongji Medical College, Huazhong University of Science and Technology, Wuhan, China; 2 Cancer Institute, Shenzhen Key Laboratory of Gastrointestinal Cancer Translational Research, Peking University Shenzhen Hospital, Shenzhen Peking University-the Hong Kong University of Science and Technology (PKU-HKUST) Medical Center, Institute of Cancer Research, Shenzhen Bay Laboratory, Shenzhen, China; 3 Department of Gastroenterology, The People’s Hospital of Jianshi, Enshi, China; 4 Department of Pediatrics, Tongji Hospital, Tongji Medical College, Huazhong University of Science and Technology, Wuhan, China

**Keywords:** microRNA-29a-3p, Roundabout homolog 1, *Schistosoma japonicum*, Hepatic fibrosis, Hepatic stellate cells, Therapy

## Abstract

**Background:**

Schistosomiasis is a serious but neglected parasitic disease in humans that may lead to liver fibrosis and death. Activated hepatic stellate cells (HSCs) are the principal effectors that promote the accumulation of extracellular matrix (ECM) proteins during hepatic fibrosis. Aberrant microRNA-29 expression is involved in the development of fibrotic diseases. However, less is known about the role of miR-29 in *Schistosoma japonicum* (*S. japonicum*)-induced hepatic fibrosis.

**Methods:**

The levels of microRNA-29a-3p (miR-29a-3p) and Roundabout homolog 1 (Robo1) were examined in liver tissues during *S. japonicum* infection. The possible involvement of the miR-29a-3p-Robo1 signaling pathway was determined. We used MIR29A conditional knock-in mice and mice injected with an miR-29a-3p agomir to investigate the role of miR-29a-3p in schistosomiasis-induced hepatic fibrosis. The functional contributions of miR-29a-3p-Robo1 signaling in liver fibrosis and HSC activation were investigated using primary mouse HSCs and the human HSC cell line LX-2.

**Results:**

MiR-29a-3p was downregulated in humans and mice with schistosome-induced fibrosis, and Robo1 was upregulated in liver tissues. The miR-29a-3p targeted Robo1 and negatively regulated its expression. Additionally, the expression level of miR-29a-3p in schistosomiasis patients was highly correlated with the portal vein and spleen thickness diameter, which represent the severity of fibrosis. Furthermore, we demonstrated that efficient and sustained elevation of miR-29a-3p reversed schistosome-induced hepatic fibrosis. Notably, we showed that miR-29a-3p targeted Robo1 in HSCs to prevent the activation of HSCs during infection.

**Conclusions:**

Our results provide experimental and clinical evidence that the miR-29a-3p-Robo1 signaling pathway in HSCs plays an important role in the development of hepatic fibrosis. Therefore, our study highlights the potential of miR-29a-3p as a therapeutic intervention for schistosomiasis and other fibrotic diseases.

**Graphical Abstract:**

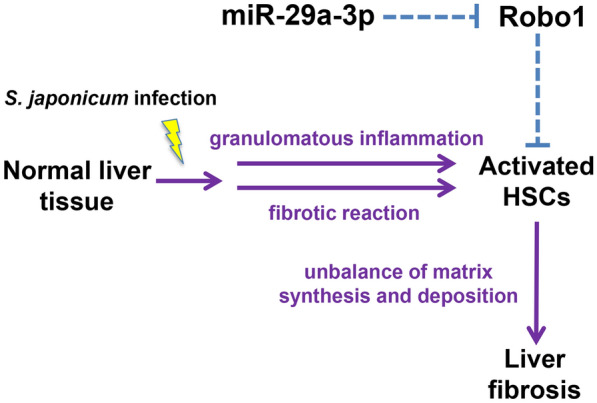

**Supplementary Information:**

The online version contains supplementary material available at 10.1186/s13071-023-05791-4.

## Background

Schistosomiasis is one of the most prevalent, but neglected, tropical infectious diseases, and it affects more than 230 million people in 78 countries, including children and young adults [1]. The two most important species that cause liver disease in humans are *Schistosoma mansoni* and *Schistosoma* *japonicum* (*S.* *japonicum*) [2]. The primary pathology of *S. japonicum*-induced schistosomiasis is egg-induced granuloma formation and fibrosis. Female adult worms living in the host mesenteric veins lay numerous eggs, most of which become trapped in the liver tissue via the portal venous system, causing a granulomatous reaction and fibrosis [3]. Hepatic fibrosis and the resulting portal hypertension are the primary causes of mortality associated with this chronic disease [4]. Elucidating the mechanisms that result in hepatic fibrosis may lead to more effective intervention strategies for schistosomiasis and a variety of fibrotic diseases.

Hepatic stellate cells (HSCs) are the main effectors of various types of hepatic fibrosis [5], including schistosome infection-induced hepatic fibrosis [6]. Quiescent HSCs are resident perisinusoidal cells located in the subendothelial space between hepatocytes and sinusoidal endothelial cells, and these cells are characterized by the presence of cytoplasmic vitamin A droplets [7]. Upon activation, HSCs gradually transform into proliferative, contractile, and fibrogenic myofibroblasts [8]. Liver injury stimulates a variety of cytokines and growth factors that activate HSCs to produce α-smooth muscle actin (α-SMA) and secrete excess extracellular matrix (ECM), which results in liver fibrosis [9, 10]. It has been shown that schistosome infection activates HSCs distributed around the periphery of egg-induced granulomas [11]. Previous studies suggested that transforming growth factor-β1 (TGF-β1) was the effector cytokine of schistosome-induced hepatic fibrosis, and it remains the classic fibrogenic cytokine driving the activation of HSCs [12–14]. Blocking the activation of HSCs has become one of the major strategies for therapeutic interventions for hepatic fibrosis [15].

MicroRNAs (miRNAs) are endogenous, small noncoding RNAs that negatively regulate gene expression by base pairing with the 3′ untranslated region of their target messenger RNA (mRNA) [16, 17]. Increasing evidence has demonstrated that miRNAs play important roles in maintaining cellular homeostasis under normal and diseased conditions [18]. The dysregulation of miRNA expression is involved in fibrosis in multiple organ systems, including the vasculature (pulmonary fibrosis), liver, and kidney [19–22]. Several studies showed that miRNAs play a crucial role in the pathogenesis of liver fibrosis and may be useful therapeutic targets [23, 24]. Our previous findings indicated that increased expression of miR-182 promoted HSC activation by targeting *FOXO1*, which resulted in the induction of hepatic fibrosis [25]. The miR-29 family consists of miR-29a, miR-29b, and miR-29c, which were significantly decreased in fibrotic livers, as demonstrated in human liver fibrosis and two different models of liver injury induced by bile duct ligation (BDL) and carbon tetrachloride (CCl_4_) [22, 26, 27]. However, there is no experimental evidence to demonstrate that miR-29a is involved in the occurrence or progression of schistosome infection-induced hepatic fibrosis.

Roundabout homolog 1 (Robo1) is a member of the neural cell adhesion molecular family of receptors, and it is expressed in various cell types [28, 29]. Robo1 is a critical regulator in multiple biological processes, including cell proliferation, differentiation, and migration [30, 31]. Robo1 is related to several types of cancer [32, 33] and chronic diseases, including kidney disease [34] and liver fibrosis [35]. Our previous results indicated that microRNA-29a-3p (miR-29a-3p) downregulated the expression of Robo1 to inhibit hepatocellular carcinoma cell proliferation, migration, invasion, tumor progression, and metastasis [36]. In light of these findings, we hypothesized that miR-29a-3p and Robo1 were critically involved in schistosomiasis-induced hepatic fibrosis. The present study used *S. japonicum* in humans and mice to investigate the role of miR-29a-3p and Robo1. We used MIR29A conditional knock-in mice and mice injected with an miR-29a-3p agomir to investigate our hypothesis and elucidate the molecular pathogenesis pathways of miR-29a-3p and Robo1 in the fibrotic changes of human and mouse livers upon exposure to schistosome infection.

## Methods

### Patients

Liver biopsy specimens were collected from Tongji Hospital, Tongji Medical College, Huazhong University of Science and Technology, between September 2015 and August 2019. Wedge biopsy specimens from normal portions of the liver were obtained from seven patients without metastatic liver carcinoma (3 colonic carcinomas and 4 gastric carcinomas) as controls. Fibrotic liver specimens were collected from nine patients with chronic schistosomiasis. The disease state of the tissues was confirmed by histopathological diagnosis. Study exclusion criteria were patients with other types of hepatitis and liver disease associated with drug or alcohol use. Unless otherwise stated, the n-values refer to the number of patients from whom tissue was obtained. Due to the limited number of tissue samples, not all samples were included in every study protocol. The demographic characteristics of the enrolled patients are summarized in Table 1.

**Table 1:** Patients’ demographic characteristics

	Control	Fibrosis	*P* value
Liver samples			
Subject no.	7	9	
Male sex	4 (57%)	5 (56%)	0.949
Age(years)	60 (59–67)	49 (40–63)	0.134
History of *Schistosoma japonicum*			
< 10 years	0 (0%)	0 (0%)	
10–20 years	0 (0%)	5 (56%)	
> 20 years	0 (0%)	4 (44%)	
Ascites	0 (0%)	5 (56%)	0.017*
EGVB	0 (0%)	4 (44%)	0.042*

For continuous variables, the results are expressed as medians and interquartile ranges. Categorical variables are summarized with percentages. *Statistically significant. EGVB: esophageal gastric variceal bleeding

### Mice

Generation of the human MIR29A conditional knock-in mouse line by CRISPR/Cas9 was outsourced to Cyagen Biosciences (Suzhou, China). The mice were created on the C57BL/6J genetic background. The gRNA (5′-GAACACTAGTGCACTTATCCTGG-3′) to the Hipp11 (H11) locus, the donor vector containing the “CAG-loxP-Stop-loxP-human MIR29A-polyA” cassette, and Cas9 mRNA were co-injected into fertilized mouse eggs to generate targeted conditional knock-in offspring. A schematic depiction of the targeting strategy is shown in Additional file 1: Fig. S1.

### Animal models of *S. japonicum*-induced hepatic fibrosis

Six-week-old female mice were used in this study. Wild-type (WT) C57BL/6J mice were obtained from Beijing Vital River Laboratory Animal Technology Co., Ltd. (Beijing, China). Human MIR29A conditional knock-in mice were constructed by Cyagen. All animals were housed under specific pathogen-free conditions with standard laboratory food and water available ad libitum. To induce infection, seven 6-week-old female MIR29A mice and seven age-matched female WT C57BL/6J mice were exposed percutaneously to 16 ± 1 *S. japonicum* cercariae (Chinese mainland strain) obtained from infected *Oncomelania hupensis* snails purchased from Nanjing Institute of Schistosomiasis Prevention and Control (Nanjing, China). Seven age- and sex-matched noninfected mice were used as controls.

### Treatment of animals

To determine whether miR-29a-3p was sufficient to reverse egg-induced hepatic fibrosis, an miR-29a-3p agomir (RiboBio, Guangzhou, China) was used. The miR-29a-3p agomir and negative control (NC) agomir were dissolved in phosphate buffer saline (PBS) according to the manufacturer’s instructions and diluted in PBS to a final bath concentration of 10 nmol/500 μl. The infected mice were treated orally with 300 mg/kg praziquantel for 2 consecutive days to kill the parasites at 6 weeks post-infection, and the mice received the miR-29a-3p agomir or NC agomir at a dose of 10 nmol per mouse or 500 μl PBS via tail vein every 4 days for 28 days. The mice were harvested 10 weeks post-infection for further analysis.

### Liver histopathology and fibrosis measurement

To estimate the egg burden, 0.2 g of each liver was digested overnight with 20 ml 4% potassium hydroxide, and the numbers of eggs were counted under a microscope. Total eggs per gram in the liver were calculated using the following formula: the number of eggs calculated × 5. The liver or spleen index was calculated with the following formula: (total weight of mouse liver or spleen/total weight of mouse body) × 100% [12, 37, 38]. The size of egg granulomas was measured in Mayer’s H&E sections using a calibrated measuring eyepiece, and the extent of fibrosis was evaluated by Masson’s trichrome staining of sections, as previously described [12]. The total fibrosis score was determined by multiplying the density and area of each granuloma (for a maximum score of 16). The hydroxyproline content in the liver was detected using a colorimetric assay kit according to the manufacturer’s instructions (Nanjing Jiancheng Bioengineering Institute, Nanjing, China).

### Serum biochemistry

Approximately 1 ml of blood was sampled from mice via eyeball extirpation. The blood was incubated for 4 h at room temperature to allow clot formation and then centrifuged (3500RPM, 10 min) to separate the serum from the clot. The serum levels of alanine aminotransferase (ALT) and aspartate aminotransferase (AST) were measured using a Siemens Advia 1650 automatic analyzer.

### Isolation of primary HSCs, hepatocytes, and Kupffer cells

The liver was initially digested in situ with 0.05% collagenase type IV at 37 °C with bath shaking for 30 min. Hepatocytes were isolated via centrifugation of the resulting cell suspension at 50×*g* for 3 min and purified via centrifugation at 50×*g* for 2 min. After hepatocytes were pelleted, the supernatant containing nonparenchymal cells was centrifuged at 450×*g* for 8 min. HSCs were isolated from nonparenchymal cells using a 15% (w/v) and 11.5% (w/v) iodixanol gradient (OptiPrep; Stemcell Technologies, Vancouver, British Columbia, Canada) at 1450×*g* for 20 min. To further purify HSCs, we depleted HSCs of Kupffer cells (KCs) using a biotin-conjugated anti-CD271 antibody (Miltenyi Biotec, Bergisch Gladbach, Germany) and anti-biotin MicroBeads (Miltenyi Biotec). The purity was > 97%, and the viability was > 95%. Representative results for the purification of HSCs are shown in Additional file 2: Fig. S2.

### Cell culture and in vitro treatment

LX-2 is a well-characterized cell line derived from human HSCs, and these cells were acquired from the Cell Collection Center of Wuhan University (China). Primary HSCs and LX-2 cells were seeded into plastic plates and cultured in DMEM (Gibco, Gaithersburg, MD, USA) supplemented with 10% fetal bovine serum (FBS; Gibco, Gaithersburg, MD, USA) and 100 μg/ml penicillin/streptomycin (Invitrogen, Carlsbad, CA, USA). Cell cultures were maintained at 37 °C in a humidified incubator at 5% CO_2_.

LX-2 cells (2.5 × 10^5^ cells/well) were plated in six-well plates with or without the presence of recombinant TGF-β1 (PeproTech). To determine the effects of miR-29a-3p in vitro, LX-2 cells were transfected with exogenous hsa-miR-29a-3p (miRBase: MIMAT0000086). The cells were transfected with 50 nM miR-29a-3p mimics, 100 nM miR-29a-3p inhibitors or their negative controls using a ribo*FECT*™ CP Transfection Kit (RiboBio, Guangzhou, China) as described previously [39]. Briefly, 10 × ribo*FECT*™ CP buffer was diluted to 1 ×. The transfection system was the mixture of 1 × ribo*FECT*™ CP buffer, ribo*FECT*™ CP reagent, and mimic (50 nM) or inhibitors (100 nM), and the mixture was transfected into LX-2 cells; 48 h after transfection, the cells of each experimental group were re-suspended, collected, and subjected to downstream experiments.

### Immunohistochemistry and immunofluorescence

Human and mouse liver samples were fixed in 4% paraformaldehyde for 24 h, embedded in paraffin, and cut into 4-µm-thick sections. For immunohistochemistry, horseradish peroxidase (HRP)-conjugated secondary antibodies (PeproTech Inc., USA) were used for immunostaining with the following primary antibodies: rabbit polyclonal anti-Robo1 (1:50, ab7279, Abcam, Cambridge, MA, USA), rabbit polyclonal anti-collagen I (1:100, 14695-1-AP, ProteinTech, Chicago, IL, USA), and rabbit monoclonal anti-α-SMA (1:500, ab108424, Abcam). For immunofluorescence staining, tissue sections were incubated with primary antibodies against Robo1 (1:50, ab7279, Abcam), α-SMA (1:200, BM0002, Boster Biological Technology, Wuhan, China), and desmin (1:100, ab227651, Abcam), followed by incubation with Alexa Fluor 594- and Alexa Fluor 488-conjugated secondary antibodies (1:200, Invitrogen, Carlsbad, CA, USA). All sections were stained with 1 μg/ml 4′,6-diamidino-2-phenylindole (DAPI; Sigma-Aldrich) to visualize cell nuclei. At least three liver sections were included in each group. The stained sections were viewed under a microscope (Nikon Eclipse Ci), and images were captured using a high-resolution digital camera (Nikon digital sight DS-FI2).

### RNA extraction and analysis

Total RNA was isolated using TRIzol reagent (Invitrogen) according to the manufacturer’s protocol. Real-time quantitative polymerase chain reaction (qPCR) was performed as previously described [40]. The expression levels of Robo1, Col1α1, Col3α1, α-SMA, and TGF-β1 were determined using the SYBR Green Master Mix Kit (Takara, Kusatsu, Japan). The expression level of miR-29a-3p was determined with a Bulge-Loop miRNA qRT-PCR Starter Kit (RiboBio, Guangzhou, China) according to the manufacturer’s protocol. Sequence-specific primers for U6 and miR-29a-3p were synthesized by RiboBio (Guangzhou, China). The endogenous controls in this study were U6 snRNA and GAPDH, and the 2^−ΔΔCt^ method was used to calculate the fold change in the expression of all mRNAs and miR-29a-3p. The human and mouse primer sequences are presented in Table 2.

**Table 2:** Primer sequences used in this study

Gene	Species	Primer sequence (5′-3′)	Size (bp)
Robo1	Human	F: CGCCCCACACCCACTATTG R: GAAGTCATCCCGAAGTATGG	237
Col1α1	Human	F: GAAGACATCCCACCAATCACC R: TCTCGTCACAGATCACGTCATC	136
Col3α1	Human	F: CTACTTCTCGCTCTGCTTCATC R: CACAGACACATATTTGGCATGG	136
α-SMA	Human	F: GTCCCACATCAGGGAGTAA R: TCGGATATTCAGCGTCAGGA	185
TGF-β1	Human	F: TGGCGATACCTCAGCAACC R: CTAAGGCGAAAGCCCTCAAT	168
GAPDH	Human	F: GGAGCGAGATCCCTCCAAAAT R: GGCTGTTGTCATACTTCTCATGG	197
Robo1	Mouse	F: GAGCCTGCTCACTTTTACCTC R: GGTCTGAAGGGTGTTCAACAAT	184
Col1α1	Mouse	F: CGCCATCAAGGTCTACTGC R: ACGGGAATCCATCGGTCA	152
Col3α1	Mouse	F: GCCCACAGCCTTCTACACCT R: GCCAGGGTCACCATTTCTC	110
α-SMA	Mouse	F: GAAGTATCCGATAGAACACG R: CTCAAACATAATATGGGTCA	185
TGF-β1	Mouse	F: TGACGTCACTGGGGGTTGTACC R: GGTTCATGTCATGGATGGTGC	183
GAPDH	Mouse	F: CCTCGTCCCGTAGACAAAATG R: TGTAGTTGAGGTCAATGAAGGG	139

### Western blotting

Total cell protein was extracted on ice with RIPA lysis buffer (Beyotime Biotechnology, Shanghai, China) in the presence of freshly added protease inhibitors (Boster Biological Technology, Wuhan, China) and quantified by BCA assay (Pierce, Cramlington, UK). A total of 30 μg/lane protein extract was separated via 10% PAGE (Beyotime Biotechnology, Shanghai, China), followed by transfer to a PVDF membrane (Millipore Corp., Billerica, MA, USA). Nonspecific binding was blocked with 5% nonfat milk in TBST. The membrane was incubated with rabbit anti-Robo1 (1:1000, ab7279, Abcam, Cambridge, MA) overnight at 4 °C. The membranes were further incubated with HRP-conjugated anti-rabbit secondary antibodies and detected using enhanced chemiluminescence (ECL; Abbkine, Redlands, CA, USA). Rabbit anti-GAPDH (1:2000, 10494-1-AP, ProteinTech, Chicago, USA) was used as an internal standard. Densitometry was performed using ImageJ software.

### Statistical analysis

The experimental results are expressed as the mean ± standard deviation (SD). Statistical significance between experimental groups was assessed using the two-tailed Student’s *t* test and one-way ANOVA with Tukey’s correction. The clinical results from patients are expressed as the median and interquartile range. Due to the skewed distributions of most patient variables, the Mann-Whitney U test and Kruskal-Wallis with Dunn’s multiple comparisons posttest were applied. For categorical data, the *χ*^2^ test or Fisher’s exact test was performed to determine differences between groups. Spearman’s rank test was used for correlations. All statistical analyses were performed with GraphPad Prism 7.0 (GraphPad Software, La Jolla, CA, USA) or SPSS 25.0 (SPSS, Chicago, IL). *P* values < 0.05 were considered significant.

## Results

### Decreased miR-29a-3p expression and increased Robo1 expression in liver tissues of patients with schistosomiasis

**Fig. 1:**
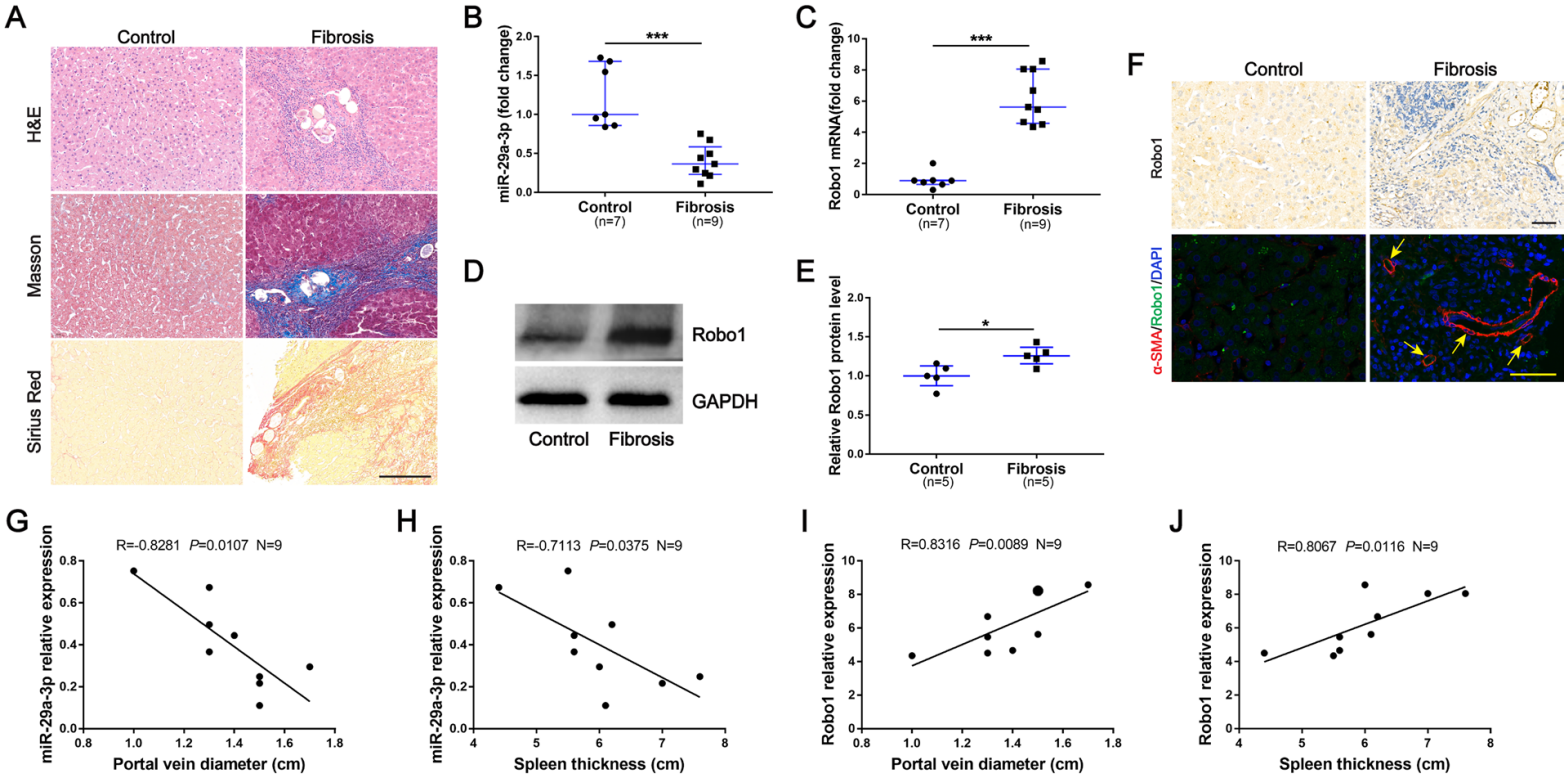
Decreased miR-29a-3p expression and increased Robo1 expression in liver tissues of patients with schistosomiasis. **A** Paraffin-embedded sections of liver tissues from patients were stained with H&E, Masson's trichrome, and Sirius Red. Scale bar, 200 μm. **B**, **C** Total RNA was extracted for qPCR analysis of miR-29a-3p and Robo1 expression levels. Control (*n* = 7), fibrosis (*n* = 9). **D**, **E** The expression of Robo1 was determined by western blotting. Image density was quantified using ImageJ and normalized to GAPDH. **F** Representative immunohistochemical staining of Robo1 and immunofluorescence staining of Robo1 (green) colocalization with α-SMA (red) in liver sections was detected. Yellow arrows denote positive cells. Scale bar, 50 μm. **G**–**J** Correlations between the mRNA levels of miR-29a-3p and Robo1 and portal vein diameter and spleen thickness in patients with schistosomiasis (*n* = 9). **I** The larger dot shows data from two patients. Data are presented as the median and interquartile range (**B**, **C** and **E**). All data are representative of at least two independent experiments. Significance was calculated using the Mann-Whitney U test (**B**, **C** and **E**) or Spearman’s rank test (**G**–**J**). *P < 0.05, ***P < 0.001. miR-29a-3p: microRNA-29a-3p; Robo1: Roundabout homolog 1

To examine the expression of miR-29a-3p and Robo1 in liver specimens, liver biopsy was performed in chronic schistosomiasis and control patients. The fibrotic liver tissues had larger fibrotic areas and more collagen deposition, and *S. japonicum* eggs were observed in the liver specimen sections of patients with schistosomiasis (Fig. 1A). The level of miR-29a-3p in the fibrotic group was significantly lower than the control group, and the level of Robo1 mRNA was increased (Fig. 1B, C). Immunoblots were analyzed, and the level of Robo1 transcription was confirmed (Fig. 1D, E). To further evaluate the functional effects of Robo1, we analyzed Robo1 in liver tissues by immunohistochemistry. The fibrotic tissues showed stronger Robo1 staining than the control liver tissues, and the Robo1-positive cells were localized primarily in areas of inflammation and fibrosis (Fig. 1F). Immunofluorescence staining was used to detect the expression of Robo1 and the presence of HSCs, which are the main effector cells in various types of hepatic fibrosis and play an important role in linking hepatic inflammation to fibrogenesis. The colocalization of Robo1 and HSCs was observed, particularly in the livers of patients with schistosomiasis (Fig. 1F). In addition, we found significant correlations between the levels of miR-29a-3p and Robo1 and portal vein diameter and spleen thickness in patients with schistosomiasis (Fig. 1G–J). These data suggested that miR-29a-3p and Robo1 may be involved in the progression of schistosome-induced hepatic fibrosis.

### miR-29a-3p is downregulated in murine schistosomiasis and directly targets and negatively regulates Robo1 in HSCs

**Fig. 2:**
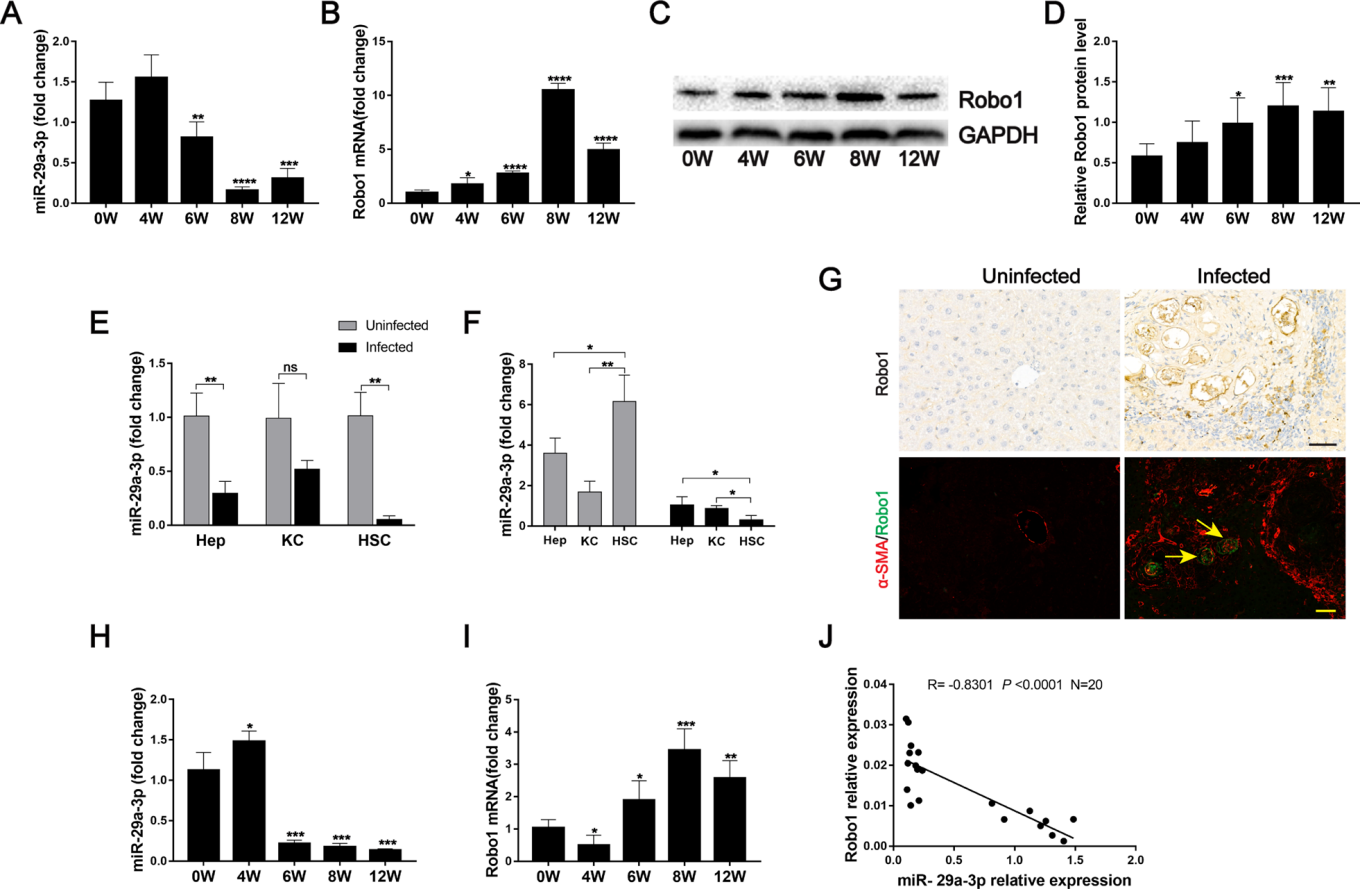
Analysis of miR-29a-3p and Robo1 expression in murine schistosomiasis. **A**, **B** The expression of miR-29a-3p and Robo1 in liver samples during infection was detected by qPCR (*n* = 4–5). **C**, **D** Robo1 protein was determined by western blotting, quantified using ImageJ, and normalized to GAPDH (*n* = 6). **E**, **F** Primary hepatocytes (Heps), Kupffer cells (KCs), and hepatic stellate cells (HSCs) were isolated from uninfected and infected (8 weeks post-infection) livers, and the level of miR-29-3p was determined by qPCR (*n* = 3). **G** Representative immunohistochemical staining of Robo1 and immunofluorescence staining of Robo1 (green) colocalization with α-SMA (red) in liver sections were detected. Yellow arrows denote positive cells. Scale bar, 50 μm. **H**, **I** Primary HSCs were isolated from liver tissues, and the expression of miR-29a-3p and Robo1 in HSCs was detected by qPCR (*n* = 4). **J** Correlations between the mRNA levels of Robo1 and miR-29a-3p in the HSCs of mice (*n* = 20). Data are presented as the mean ± SD from two independent experiments. Significance was determined by the two-tailed Student’s *t* test (**A**, **B**, **D**, **E**, **H**, and **I**) or one-way ANOVA with Tukey’s correction for comparisons between two groups (**F**) or Spearman’s rank test (**J**). *P < 0.05, **P < 0.01, ***P < 0.001, ****P < 0.0001, compared with 0W samples (**A**, **B**, **D**, **H**, and **I**) . miR-29a-3p: microRNA-29a-3p; Robo1: Roundabout homolog 1; Heps: hepatocytes; KCs: Kupffer cells; HSCs: hepatic stellate cells; ns: not significant

Liver samples from mice at various time points after *S. japonicum* infection were collected. Robo1 is a potential target of miR-29a-3p, as predicted by the TargetScan database (www.targetscan.org). To analyze the relationship between miR-29a-3p and Robo1, we evaluated their expression during the progression of hepatic schistosomiasis. We found that the expression of miR-29a-3p began to decrease in the liver at 6 weeks post-infection and reached its lowest levels at weeks 8 and 12 (Fig. 2A). In contrast, the expression level of Robo1 was significantly elevated at 8 weeks post-infection (Fig. 2B–D). In addition, miR-29a-3p downregulation was primarily observed in isolated hepatocytes and HSCs of infected mice (Fig. 2E). We also analyzed the relative expression of miR-29a-3p in different hepatic cell types by qPCR and found that miR-29a-3p was primarily present in isolated primary HSCs rather than in hepatocytes or KCs of uninfected livers. The miR-29a-3p expression level was significantly downregulated in HSCs from infected livers compare to hepatocytes and KCs (Fig. 2F). We also performed immunochemistry staining for Robo1 and observed that Robo1-producing cells were primarily located in the periphery of egg granulomas (Fig. 2G). Immunofluorescence double-staining revealed the colocalization of Robo1 and α-SMA (Fig. 2G), indicating that activated HSCs expressed Robo1 in vivo. The expression of miR-29-3p in HSCs began to decrease at 6 weeks post-infection, but the level of Robo1 mRNA was increased at this time (Fig. 2H, I). In addition, a negative correlation was observed between miR-29a-3p and Robo1 expression by Spearman’s correlation analysis (*R* = − 0.8301, *P* < 0.0001; Fig. 2J). Taken together, these results suggest that the activated HSCs in infected livers are a source of Robo1, and Robo1 is a potential target of miR-29a-3p in HSCs.

**Fig. 3:**
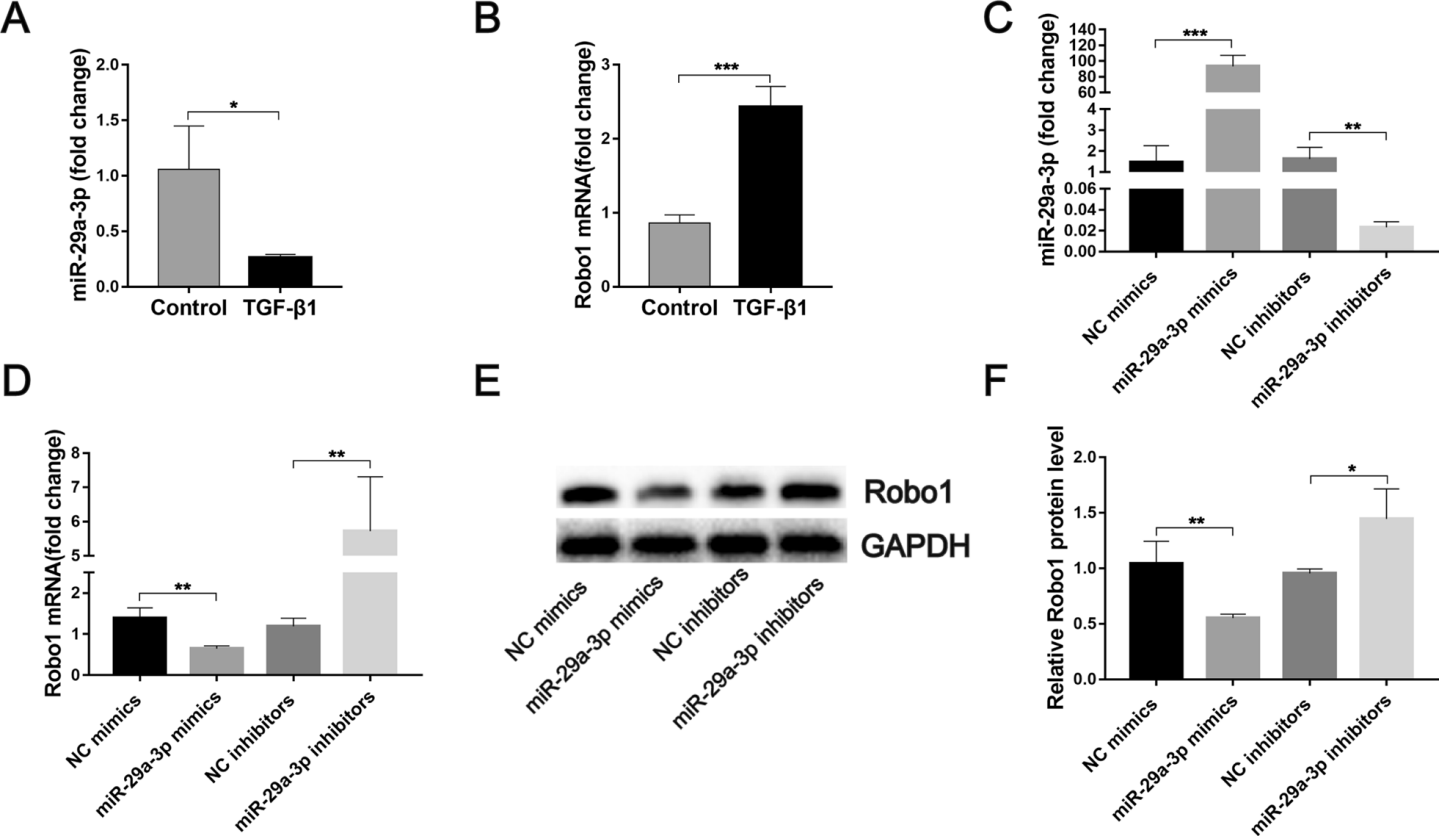
Validation of the relationship between miR-29a-3p and Robo1. **A**, **B** LX-2 cells were exposed to 10 ng/ml TGF-β1 for 48 h, and the expression of miR-29a-3p and Robo1 was detected by qPCR (*n* = 3). **C**–**F** LX-2 cells were cultured on a plastic plate and transfected with 50 nM miR-29a-3p mimics, 100 nM miR-29a-3p inhibitors, or their negative controls (NC) for 48 h. The expression of miR-29a-3p and Robo1 was determined by qPCR (*n* = 3) (**C**, **D**) and western blotting (*n* = 3–4) (**E**, **F**). Data are presented as the mean ± SD. All data are representative of at least three independent experiments. Significance was determined by the two-tailed Student’s *t* test (**A**–**D**, **F**). *P < 0.05, **P < 0.01, ***P < 0.001. miR-29a-3p: microRNA-29a-3p; Robo1: Roundabout homolog 1

Our previous study used a dual-luciferase reporter gene assay to demonstrate that Robo1 was a direct target of miR-29a-3p [36]. The present study showed that stimulation of LX-2 cells with TGF-β1, which is a typical profibrotic gene in the progression of hepatic fibrosis, downregulated the mRNA expression of miR-29a-3p and upregulated the level of Robo1 mRNA (Fig. 3A, B). Furthermore, we transfected miR-29a-3p mimics or inhibitors into LX-2 cells and quantified the levels of miR-29a-3p and Robo1 by qPCR and western blotting. As expected, miR-29a-3p was elevated in the miR-mimic group and reduced in the miR-inhibitor group (Fig. 3C). At both the mRNA and protein levels, the elevation of miR-29a-3p distinctly reduced the expression of Robo1, and miR-29a-3p depletion significantly increased the expression of Robo1 (Fig. 3D–F). Taken together, these data indicate that Robo1 is a direct target of miR-29a-3p in HSCs.

### MIR29A mice develop liver injury and fibrosis less readily during schistosome infection

**Fig. 4:**
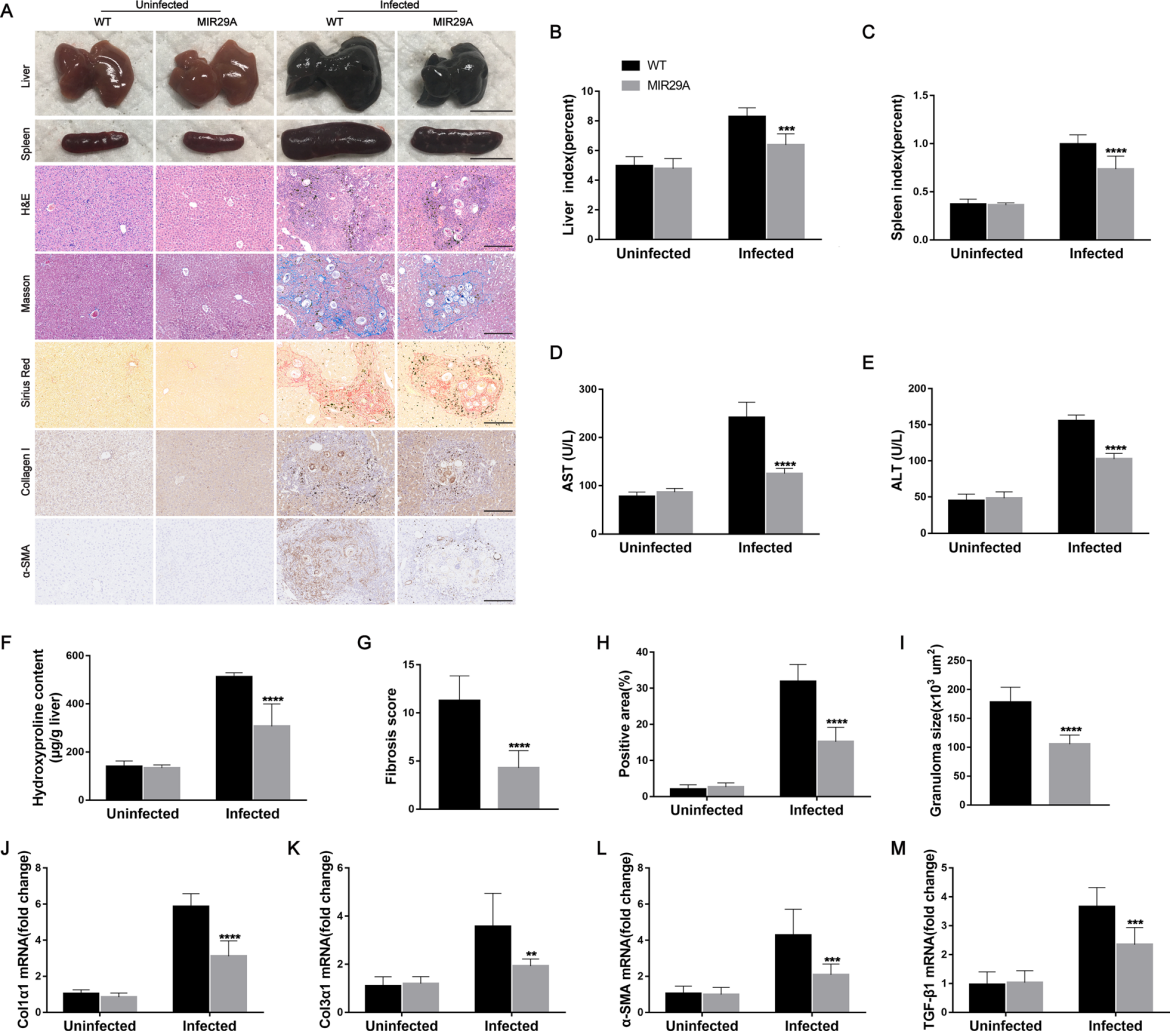
MIR29A mice developed liver injury and fibrosis less readily during schistosome infection. MIR29A mice and WT mice were infected percutaneously with 16 *Schistosoma japonicum* cercariae or remained uninfected. Liver and spleen samples were harvested at 10 weeks post-infection. **A** Macrograph of livers and spleens from MIR29A mice and WT mice in both the uninfected and infected groups. Scale bar, 1 cm. H&E, Masson's trichrome and Sirius Red staining of liver sections and immunohistochemical staining for collagen I and α-SMA. Scale bar, 50 μm. **B**, **C** Liver and spleen indices were determined (*n* = 7). **D**, **E** Serum ALT and AST levels were measured (*n* = 7). **F** Collagen content in livers determined as the hydroxyproline content (*n* = 7). **G** Fibrosis scores measured from Masson's trichrome staining of liver sections (*n* = 7). **H** Areas positive for Sirius Red staining were measured using IPP software (*n* = 7). **I** Granuloma size was measured from H&E-stained liver sections (*n* = 7). **J**–**M** The expression of Col1α1, Col3α1, α-SMA, and TGF-β1 in livers during infection was detected by qPCR (*n* = 7). Data are presented as the mean ± SD of three independent experiments. Multiple comparisons were performed by one-way ANOVA with Tukey’s correction for comparisons between two groups (B-M). **P < 0.01, ***P < 0.001, ****P < 0.0001, compared with infected WT samples. miR-29a-3p: microRNA-29a-3p; Robo1: Roundabout homolog 1; ALT: alanine aminotransferase; AST: aspartate aminotransferase

To evaluate the effects of miR-29a-3p-Robo1 signaling on the pathogenesis of liver fibrosis, we established a model of *S. japonicum*-induced hepatic fibrogenesis in human MIR29A conditional knock-in mice. WT mice underwent the same treatment as controls. Compared with WT mice, MIR29A mice showed a higher level of miR-29a-3p in the heart (approximately 2.0-fold), liver (approximately 1.8-fold), spleen (approximately 3.2-fold), and kidney (approximately 2.2-fold) (Additional file 3: Fig. S3). Morphological changes in liver and spleen samples showed a moderate granulomatous response and splenomegaly in the *S. japonicum*-infected MIR29A mice compared with the infected WT mice (Fig. 4A). These results were confirmed by the reduced levels of alanine aminotransferase (ALT) and aspartate aminotransferase (AST) in mouse serum and further confirmed by the liver and spleen indices (Fig. 4B–E). Notably, the *S. japonicum*-infected MIR29A mice exhibited a significant reduction in ECM deposits, as shown by hydroxyproline quantification (Fig. 4F), Masson’s trichrome staining, and Sirius Red staining (Fig. 4A, G and H), and a marked reduction in the size of hepatic granulomas as visualized by H&E staining (Fig. 4A, I). A reduction in fibrosis was further confirmed by immunohistochemical staining and qPCR-based quantification of fibrosis-associated gene expression in the livers of infected mice. Immunohistochemical staining for collagen I and α-SMA and the amounts of Col1α1, Col3α1, α-SMA, and TGF-β1 mRNA were markedly reduced in the *S. japonicum*-infected MIR29A mice compared with the infected WT mice (Fig. 4A, J–M). However, there was no significant change in egg burden between the two infected groups, which indicated that miR-29a treatment had no effect on parasite reproduction and survival (Additional file 4: Fig. S4A).

**Fig. 5:**
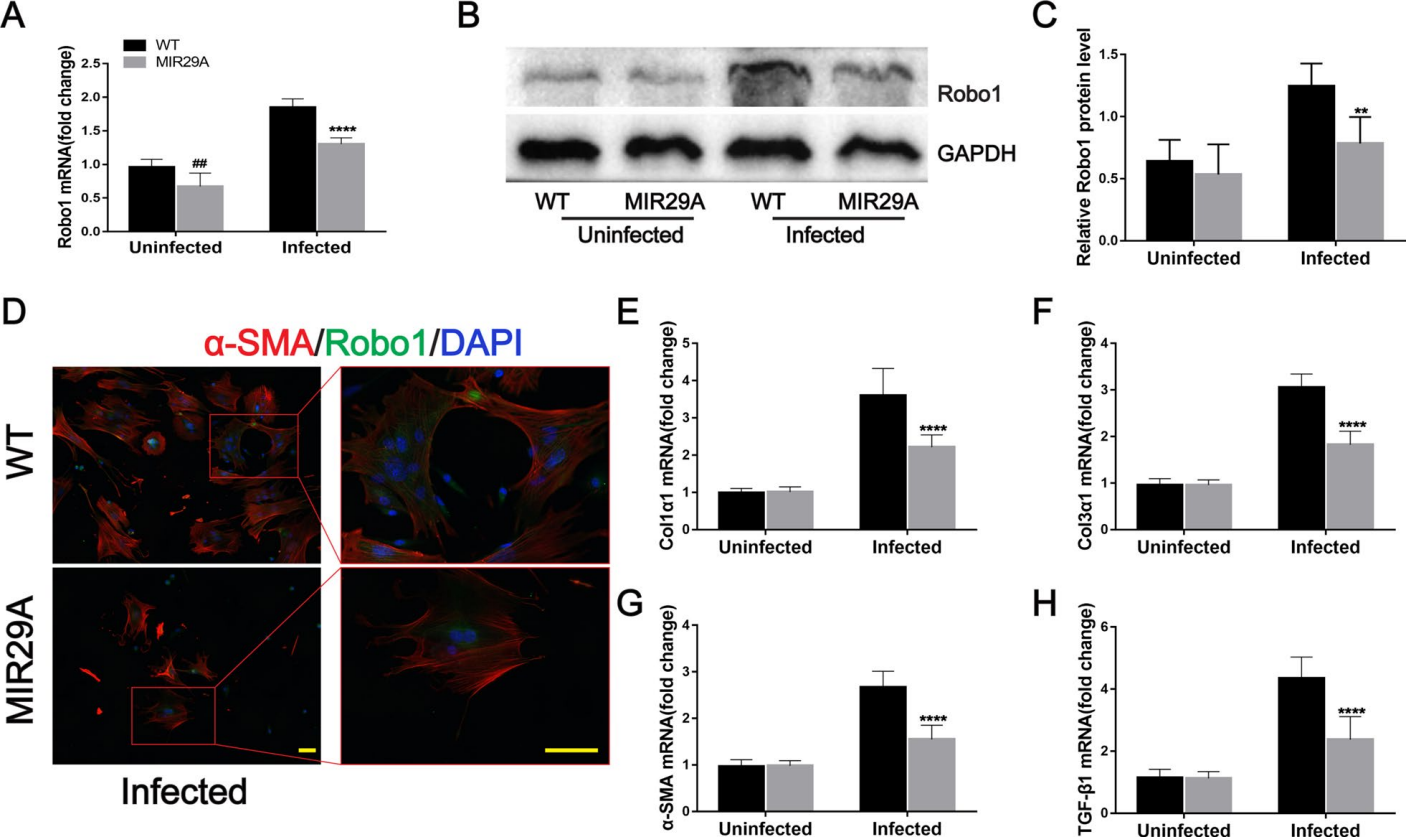
MIR29A mice were better able to prevent HSC activation during schistosome infection. **A** The expression level of Robo1 in livers was determined by qPCR (*n* = 7). **B**, **C** Robo1 protein was determined by western blotting, quantified using ImageJ, and normalized to GAPDH (*n* = 6). **D**–**H** Primary HSCs were isolated from the liver tissues of mice. **D** Representative immunofluorescence staining of Robo1 (green) colocalization with α-SMA (red) in primary HSCs was detected. *Insets* show a higher magnification of the outlined area. Scale bar, 50 μm. **E**–**H** The expression of Col1α1, Col3α1, α-SMA, and TGF-β1 in primary HSCs during infection was detected by qPCR (*n* = 7). Data are presented as the mean ± SD from two independent experiments. Significance was determined by one-way ANOVA with Tukey’s correction for comparisons between two groups (**A**, **C**, **E**–**H**). ^##^*P* < 0.01, compared with uninfected WT samples. **P < 0.01, ****P < 0.0001, compared with infected WT samples. miR-29a-3p: microRNA-29a-3p; Robo1: Roundabout homolog 1

We next evaluated the level of Robo1 in these mice. Robo1 mRNA expression was significantly lower in the liver tissues of MIR29A mice than in WT mice in the uninfected and infected groups (Fig. 5A). Robo1 protein was decreased in the liver tissues of *S. japonicum*-infected MIR29A mice compared with infected WT mice, as determined by western blotting. Although the uninfected MIR29A mouse group exhibited a slight reduction in Robo1 expression compared with the uninfected WT mouse group, the difference was not statistically significant (Fig. 5B, C). We next investigated whether the overexpression of miR-29a prevented schistosomiasis-induced HSC activation. We isolated primary HSCs from mice and found that HSCs in infected mouse liver tissues expressed Robo1 in vivo based on immunofluorescence staining. Furthermore, the fluorescence intensity of Robo1 was decreased in the infected MIR29A mice compared with the infected WT mice (Fig. 5D). The amounts of Col1α1, Col3α1, α-SMA, and TGF-β1 mRNA were markedly reduced in the infected HSCs of MIR29A mice compared with WT mice (Fig. 5E–H). These results suggested that miR-29a overexpression reduced the expression of Robo1 and prevented schistosomiasis-induced HSC activation.

### miR-29a-3p agomir-mediated elevation of miR-29a-3p has therapeutic potential for schistosome infection-induced hepatic fibrosis

**Fig. 6:**
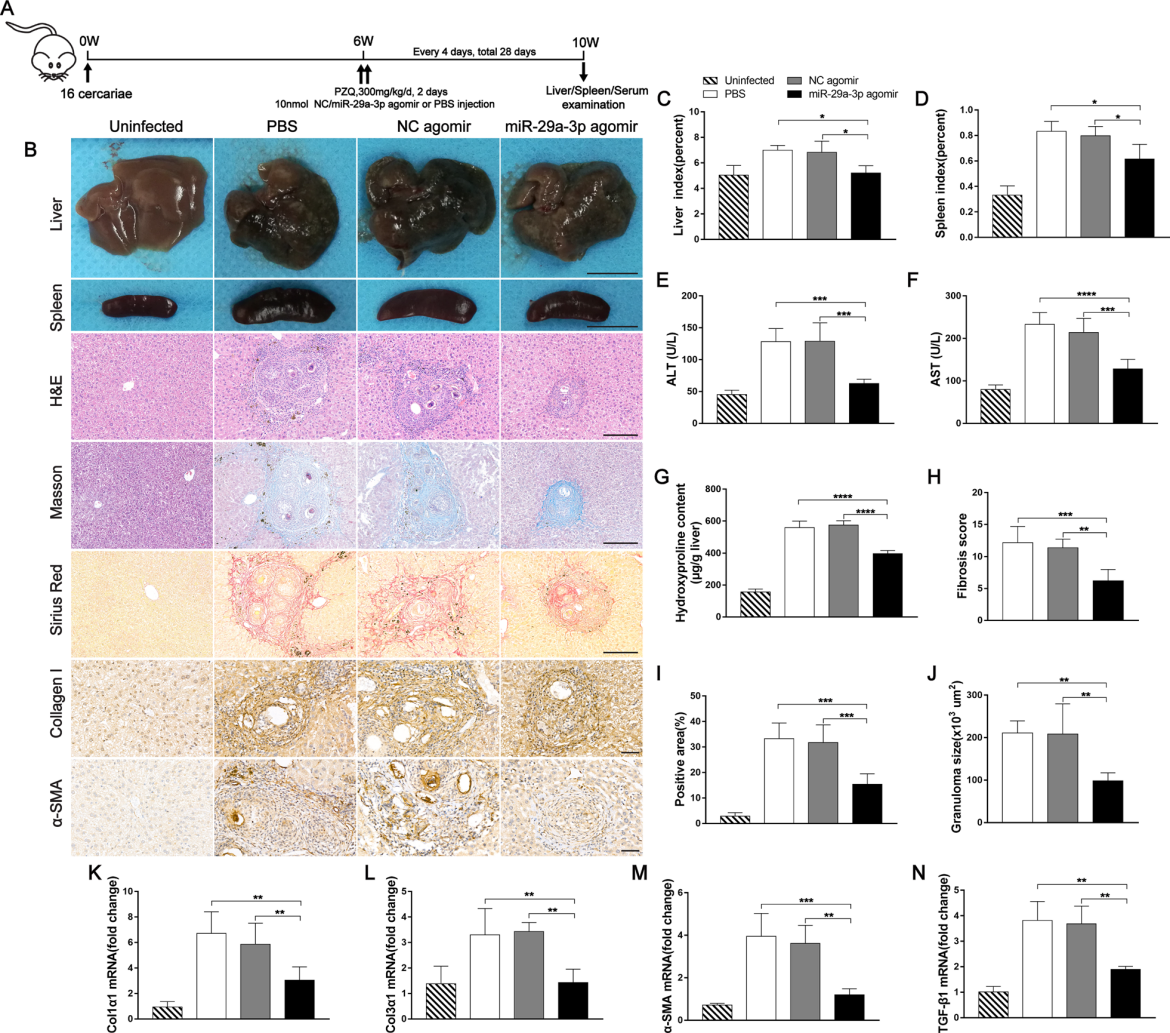
miR-29a-3p agomir-mediated elevation of miR-29a-3p partially reversed schistosome-induced hepatic fibrosis. Mice were infected percutaneously with 16 *Schistosoma japonicum* cercariae or remained uninfected. At 6 weeks post-infection, the infected mice were treated with praziquantel to kill the parasites and then received miR-29a-3p agomir, NC agomir, or PBS every 4 days for 28 days. Mice were necropsied at 10 weeks post-infection. **A** Time schedule for parasite infection and administration of anti-parasite drug or miR-29a-3p agomir and sample withdrawal. **B** Macrographs of livers and spleens from uninfected mice, infected mice, and infected mice treated with miR-29a-3p agomir. Scale bar, 1 cm. H&E, Masson's trichrome, and Sirius Red staining of liver sections and immunohistochemical staining for collagen I and α-SMA. Scale bar, 50 μm. **C**, **D** Liver and spleen indices were determined (*n* = 3–5). **E**, **F** Serum ALT and AST levels were measured (*n* = 5). **G** Collagen content in livers was determined by the hydroxyproline content (*n* = 4–5). **H** Fibrosis scores measured from Masson's trichrome staining of liver sections (*n* = 5). **I** Areas positive for Sirius Red staining were measured using IPP software (*n* = 5). **J** Granuloma size measured from H&E-stained liver sections (*n* = 5). **K**–**N** The expression of Col1α1, Col3α1, α-SMA, and TGF-β1 in livers during infection was detected by qPCR (*n* = 4). Data are presented as the mean ± SD of three independent experiments. Multiple comparisons were performed by one-way ANOVA with Tukey’s correction for comparisons between two groups (**C**–**N**). *P < 0.05, **P < 0.01, ***P < 0.001, ****P < 0.0001. miR-29a-3p: microRNA-29a-3p; Robo1: Roundabout homolog 1; ALT: alanine aminotransferase; AST: aspartate aminotransferase

To verify whether miR-29a-3p contributed to reversing egg-induced hepatic fibrosis, mice were infected with a moderate dose of parasites. At 6 weeks post-infection, when hepatic fibrosis was clearly manifested, all infected mice were treated with praziquantel to kill the parasite and then injected with miR-29a-3p agomir, NC agomir, or PBS every 4 days for 28 days and necropsied at 10 weeks post-infection (Fig. 6A). Liver and spleen samples from the miR-29a-3p agomir-treated group exhibited a moderate granulomatous response and splenomegaly (Fig. 6B). Antifibrosis treatment significantly reduced the liver and spleen indices (Fig. 6C, D). Furthermore, the mice treated with the miR-29a-3p agomir showed significant reductions in circulating levels of ALT and AST, suggesting that the treatment alleviated hepatocellular damage (Fig. 6E, F). Mice that received the miR-29a-3p agomir exhibited a significant reduction in ECM deposition, as shown by hydroxyproline quantification (Fig. 6G), Masson’s trichrome staining, and Sirius Red staining (Fig. 6B, H and I), and a marked reduction in the size of hepatic granulomas, as visualized on H&E staining (Fig. 6B, J). However, the livers showed no significant change in egg burden among the three infected groups (Additional file 4: Fig. S4B). Consistently, representative immunohistochemical staining and qPCR analysis demonstrated reduced expression levels of fibrosis markers in the livers of mice treated with the miR-29a-3p agomir (Fig. 6B, K–N).

**Fig. 7:**
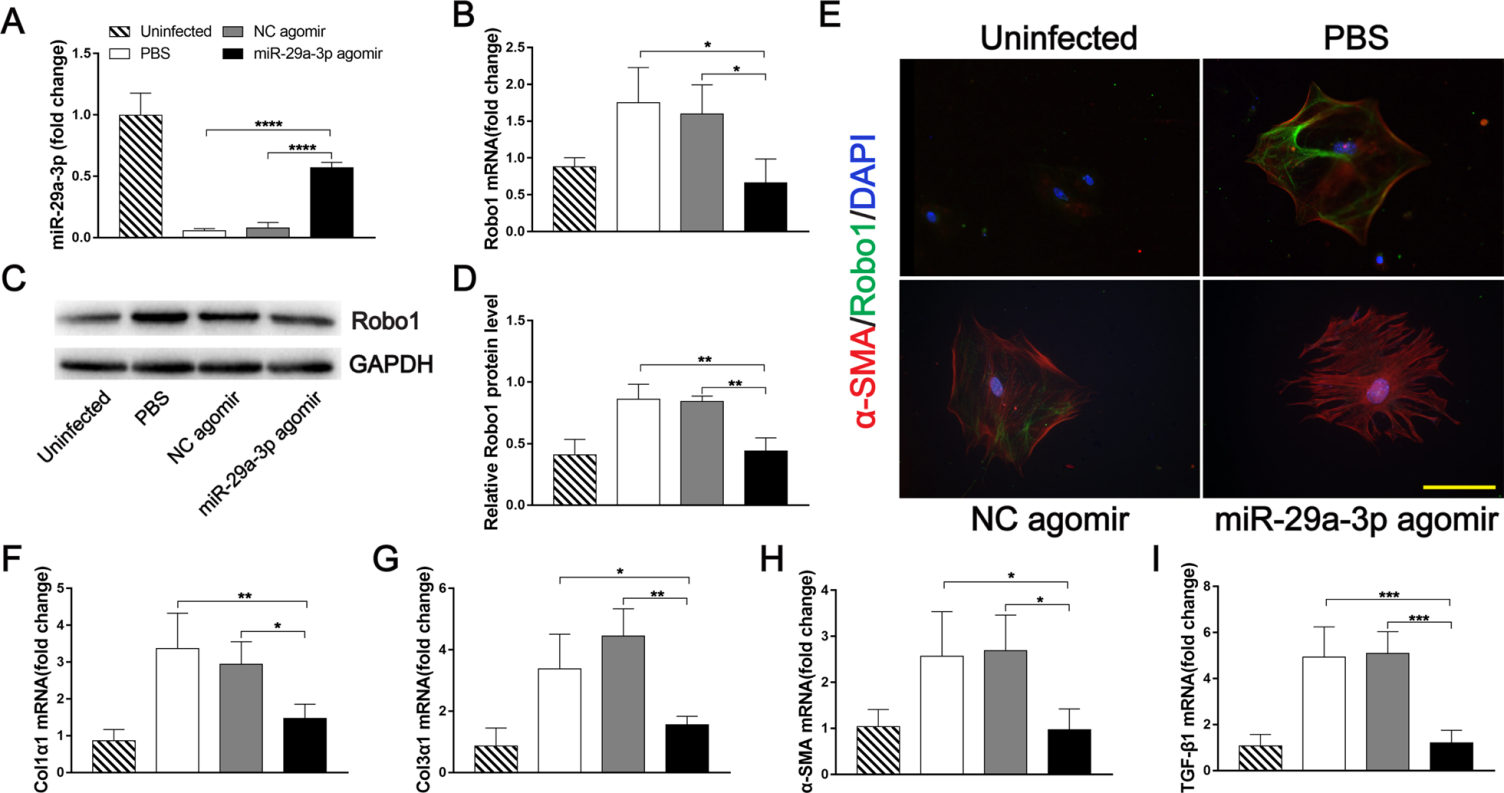
miR-29a-3p agomir-mediated elevation of miR-29a-3p prevented HSC activation during schistosome infection. **A**, **B** miR-29a-3p and Robo1 expression levels in livers were determined by qPCR (*n* = 3–4). **C**, **D** Robo1 protein was determined by western blotting, quantified using ImageJ, and normalized to GAPDH (*n* = 3). **E**–**I** Primary HSCs were isolated from the liver tissues of mice. **E** Representative immunofluorescence staining of Robo1 (green) colocalization with α-SMA (red) in primary HSCs. Scale bar, 100 μm. **F**–**I** The expression of Col1α1, Col3α1, α-SMA, and TGF-β1 in primary HSCs during infection was detected by qPCR (*n* = 3–4). Data are presented as the mean ± SD from two independent experiments. Significance was determined by one-way ANOVA with Tukey’s correction for comparisons between two groups (**A**, **B**, **D**, **F**–**I**). *P < 0.05, **P < 0.01, ***P < 0.001, ****P < 0.0001. miR-29a-3p: microRNA-29a-3p; Robo1: Roundabout homolog 1

As expected, significantly elevated miR-29a-3p expression was observed in the infected mice treated with miR-29a-3p agomir compared with the NC agomir and PBS groups (7.2- to 9.9-fold) (Fig. 7A), which resulted in markedly decreased Robo1 expression, as determined by qPCR and western blotting (Fig. 7B–D). Similar to that in MIR29A mice, colocalization of Robo1 and α-SMA+ HSCs was detected in the livers of infected mice. Meanwhile, the Robo1 expression in HSCs of infected mice treated with miR-29a-3p agomir was lower than in the infected mice treated with the NC agomir or PBS (Fig. 7E). The mRNA levels of Col1α1, Col3α1, α-SMA, and TGF-β1 were also downregulated in the infected HSCs of mice treated with the miR-29a-3p agomir (Fig. 7F–I). Taken together, these data demonstrated that miR-29a-3p upregulation reduced Robo1 expression and prevented schistosomiasis-induced HSC activation.

### Activation of miR-29a-3p-Robo1 signaling mediates HSC activation in vitro

**Fig. 8:**
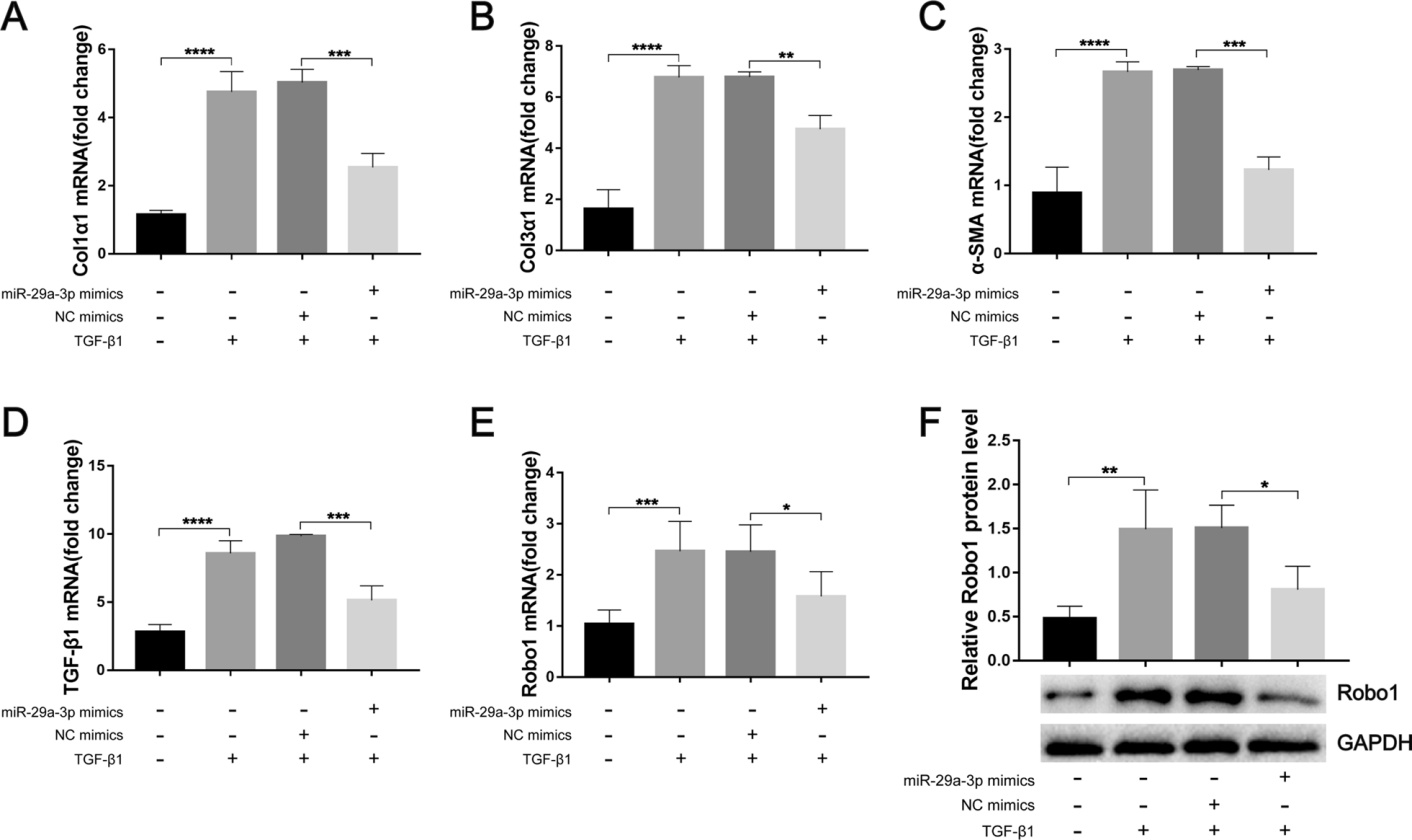
Overexpression of miR-29a-3p attenuated HSC activation by inhibiting Robo1 pathways. LX-2 cells were pretreated with 50 nM miR-29a-3p mimics or their negative controls for 6 h, TGF-β1 (10 ng/ml) was added to the medium, and the expression levels of fibrosis markers and Robo1 were determined after 48 h of incubation. **A**–**E** The expression of Col1α1, Col3α1, α-SMA, TGF-β1, and Robo1 in LX-2 cells was detected by qPCR (*n* = 3–6). **F** Robo1 protein was determined by western blotting, quantified using ImageJ, and normalized to GAPDH (*n* = 4). Data are presented as the mean ± SD of three independent experiments. Multiple comparisons were performed by one-way ANOVA with Tukey’s correction for comparisons between two groups (**A**–**F**). *P < 0.05, **P < 0.01, ***P < 0.001, ****P < 0.0001. miR-29a-3p: microRNA-29a-3p; Robo1: Roundabout homolog 1

We next investigated whether miR-29a-3p prevented TGF-β1-induced HSC activation. Using a well-validated immortalized human HSC cell line (LX-2), we found that the overexpression of miR-29a-3p significantly attenuated the mRNA levels of Col1α1, Col3α1, α-SMA, and TGF-β1 in LX-2 cells in the presence of TGF-β1 (Fig. 8A–D). Furthermore, miR-29a-3p mimics markedly downregulated Robo1 in LX-2 cells in the presence of TGF-β1 (Fig. 8E, F). These results demonstrated that miR-29a-3p overexpression attenuated HSC activation by inhibiting Robo1 pathways both in vivo and in vitro.

## Discussion

It has been previously suggested that the regulation and function of miRNAs is highly organ- and cell-type specific [41]. HSCs are the main source of myofibroblasts in the liver [5]. Our findings provide evidence for a functional role of miR-29a-3p in human and murine schistosome infection-induced hepatic fibrosis. Our data showed that miR-29a-3p was downregulated in HSCs during hepatic fibrosis, and Robo1 was upregulated. Notably, our findings suggest that miR-29a-3p partially reverses schistosome-induced hepatic fibrosis by targeting Robo1 to prevent schistosomiasis-induced HSC activation during infection.

MiR-29 family members are considered fibromiRs and have been shown to be dysregulated during the process of fibrosis in multiple organs [42]. These molecules regulate multiple collagen isoforms and other ECM components [43]. In general, decreased expression of miR-29 family members is associated with increased ECM deposition and fibrosis in various tissues. The role of miR-29 species was widely studied in animal models and patient biopsies of liver fibrosis [17, 26, 27]. However, no study to date has examined the role of miR-29 in hepatic fibrosis caused by schistosomiasis. In the present study, we found that miR-29a-3p was decreased in liver tissues with schistosomiasis-induced hepatic fibrosis. Previous studies identified that Robo1, as a potential target of miR-29a-3p, was a TGF-β1 target gene in mammary cells [44], which indicated that Robo1 may be involved in the progression of hepatic fibrosis. Meanwhile, we examined the expression of Robo1 in control and schistosomiasis patients. Our results showed that the expression level of Robo1 was significantly higher in liver tissues with hepatic fibrosis. In addition, the mRNA levels of miR-29a-3p and Robo1 in the liver tissues of fibrosis patients were related to portal vein diameter and spleen thickness, which increased in parallel with the extent of hepatic fibrosis [45, 46]. More importantly, we showed that Robo1-producing cells were primarily located in HSCs. We further found that Robo1 expression in HSCs was significantly increased after infection. These results suggest that miR-29a-3p and Robo1 are involved in the pathogenesis of liver fibrosis via HSCs during schistosome infection.

To further confirm the roles of miR-29a-3p and Robo1 in vivo, we established a murine model of *S. japonicum* infection based on information from previous studies [6, 47]. Compared with the uninfected group, the schistosome-infected mice showed lower miR-29a-3p and higher Robo1 expression in liver tissues, and these expression levels were associated with the advanced stage of liver fibrosis. In addition, the miR-29a-3p expression level was significantly downregulated in HSCs of infected mice relative to hepatocytes and KCs. Consistently, our data also indicated that Robo1 production primarily co-localized with HSCs during schistosome infection. Furthermore, the targeting relationship between miR-29a-3p and Robo1 was confirmed using primary mouse HSCs and the human HSC cell line LX-2. These results support our functional data on the role of miR-29a-3p-Robo1 signaling in HSCs and suggest that this signaling is involved in the pathogenesis of liver fibrosis via HSCs during schistosome infection.

The abnormal activation of HSCs has long been established as a critical event during the pathogenesis of hepatic fibrosis [5]. Previous studies demonstrated the presence of activated HSCs in the periphery of *S. japonicum* egg granulomas in murine and human infections, and these cells are likely effector cells that contribute to granuloma-associated fibrosis, which emphasizes the importance of stellate cell modulators in the progression of hepatic schistosomiasis [6]. Increasing evidence suggests that miR-29a has antifibrotic activity. Huang et al. reported that miR-29a-overexpressing transgenic mice ameliorated liver fibrosis by modulating the profibrogenic phenotype of HSCs after BDL or a high-fat diet [48, 49]. One promising study demonstrated accelerated recovery from CCl_4_-induced liver fibrosis following miR-29a administration via the tail vein [50]. Our study demonstrated that the overexpression of miR-29a-3p in schistosome-infected mice significantly hindered hepatocellular damage and hepatic fibrosis. Furthermore, miR-29a-3p overexpression significantly reduced Robo1 expression in activated HSCs and lessened schistosomiasis-induced HSC activation. TGF-β1 is the most potent pro-fibrogenic cytokine [51]. Previous studies identified that TGF-β1 played an important role in schistosomiasis pathogenesis [52]. To investigate the interaction between miR-29a-3p and Robo1 and the effect of this interaction on HSC activation, we transfected an miR-29a-3p agomir into LX-2 cells, followed by exposure to TGF-β1. As expected, we found that the overexpression of miR-29a-3p significantly decreased TGF-β1-induced Robo1 and collagen expression. These data collectively demonstrated that miR-29a-3p-Robo1 signaling in HSCs mediated the pathogenesis of hepatic fibrosis, and miR-29a-3p overexpression had a beneficial effect on schistosome-induced hepatic fibrosis by reducing Robo1 expression and preventing HSC activation during infection.

As illustrated above, our results provide both experimental and clinical evidence that the miR-29a-3p-Robo1 signaling pathway in HSCs plays an important role in the development of hepatic fibrosis and strongly suggest that miR-29a-3p overexpression can reverse schistosome-induced hepatic fibrosis by suppressing HSC activation during infection. Although praziquantel can effectively target and kill schistosomes, the progression of hepatic fibrosis persists [53]. To date, there are no approved antifibrotic therapies. Our data clearly demonstrated that miR-29a-3p overexpression alleviated such a hepatic fibrosis by suppressing HSC activation. Therefore, miR-29a-3p constitutes a promising candidate for the development of therapeutic tools to prevent or treat hepatic fibrosis. However, further studies are clearly warranted.

## Conclusions

Our results revealed that miR-29a-3p-Robo1 signaling in HSCs mediated the pathogenesis of hepatic fibrosis, and overexpression of miR-29a-3p had a beneficial effect on schistosome-induced hepatic fibrosis by reducing the expression of Robo1 and preventing the activation of HSCs during infection. Therefore, our study provides insights into the mechanisms of miR-29a-3p regulation of schistosomiasis hepatic fibrosis and highlights the potential of miR-29a-3p as a therapeutic intervention for fibrotic diseases.

## Supplementary Information

**Additional file 1: Figure S1.** Schematic diagram of MIR29A mouse construction. The Hipp11 (H11) locus is located within an intergenic region between the Eif4enif1 and Drg1 genes on mouse chromosome 11. The human MIR29A gene (NCBI ReferenceSequence: NR_029503.1) is located on human chromosome 7. For the conditional knock-in model, the “CAG-loxP-Stop-loxP-human MIR29A-polyA” cassette was inserted into the H11 locus (~ 0.7 kb 5' of the Eif4enif1 gene and ~ 4.5 kb 3' of the Drg1gene). Cas9 and gRNA were co-injected into fertilized eggs with a targeting vector for mouse production. The pups were genotyped by PCR followed by sequencing analysis.

**Additional file 2: Figure S2.** Purity of isolated HSCs. (A, B) Representative results for HSC purification by flow cytometry and immunofluorescence. *Insets* show a higher magnification of the outlined area. Scale bar, 50 μm.

**Additional file 3: Figure S3.** MIR29A mice had higher levels of miR-29a-3p in different organs. The miR-29a-3p expression levels in different organs of WT mice and MIR29A mice were determined by qPCR. Data are presented as the mean ± SD of three independent experiments. Significance was determined by the two-tailed Student’s *t* test. ***P < 0.001, ****P < 0.0001. miR-29a-3p: microRNA-29a-3p.

**Additional file 4: Figure S4.** Alteration of parasite burden in the different groups during schistosome infection.Alteration of parasite burden in the infected WT mice and MIR29A mice.Alteration of parasite burden after administration of the miR-29a-3p agomir. Data are presented as the mean ± SD of three independent experiments. Significance was determined by the two-tailed Student’s *t* test (A) or one-way ANOVA with Tukey’s correction for comparisons between two groups (B). miR-29a-3p: microRNA-29a-3p.

## Data Availability

All data supporting the conclusions of this study are included in the article.

## Abbreviations

ALT: Alanine aminotransferase
AST: Aspartate aminotransferase
α-SMA: α-Smooth muscle actin
BDL: Bile duct ligation
CCl_4_: Carbon tetrachloride
ECM: Extracellular matrix
FBS: Fetal bovine serum
Heps: Hepatocytes
HSCs: Hepatic stellate cells
KCs: Kupffer cells
miRNAs: MicroRNAs
miR-29a-3p: MicroRNA-29a-3p
mRNA: Messenger RNA PBS Phosphate buffer saline
PBS: Phosphate buffer saline
Robo1: Roundabout homolog 1
*S. japonicum*: *Schistosoma japonicum*
TGF-β1: Transforming growth factor-β1
