# Supplementary figures and images for: MicroRNA-29a-3p prevents Schistosoma japonicum-induced liver fibrosis by targeting Roundabout homolog 1 in hepatic stellate cells
Source: Parasit Vectors. 2023 Jun 6;16:184. doi: 10.1186/s13071-023-05791-4 (PMC10245502; doi:10.1186/s13071-023-05791-4)

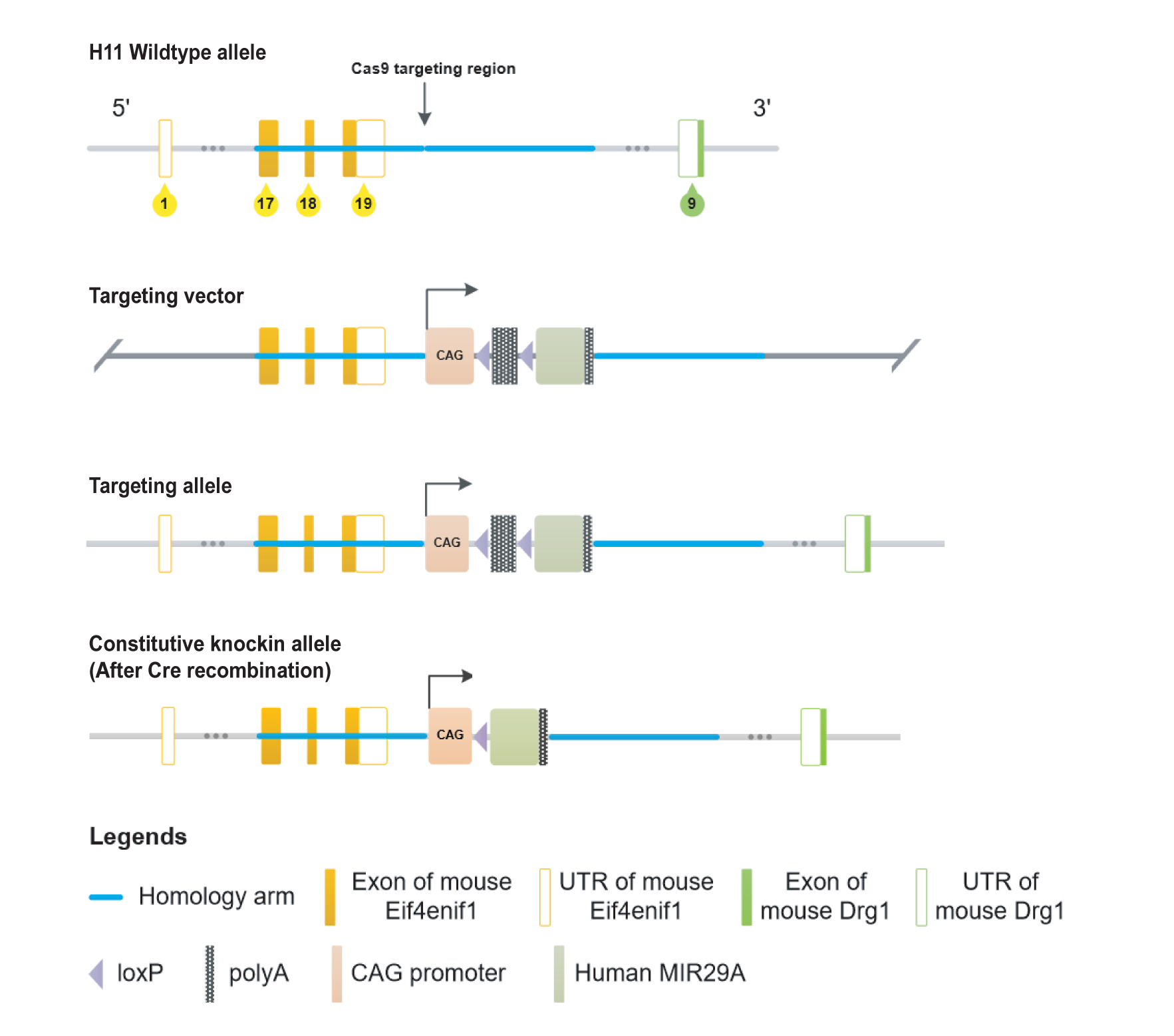

Supplement: Supplementary file 1 — Additional file 1: Figure S1. Schematic diagram of MIR29A mouse construction. The Hipp11 (H11) locus is located within an intergenic region between the Eif4enif1 and Drg1 genes on mouse chromosome 11. The human MIR29A gene (NCBI ReferenceSequence: NR_029503.1) is located on human chromosome 7. For the conditional knock-in model, the “CAG-loxP-Stop-loxP-human MIR29A-polyA” cassette was inserted into the H11 locus (~ 0.7 kb 5' of the Eif4enif1 gene and ~ 4.5 kb 3' of the Drg1gene). Cas9 and gRNA were co-injected into fertilized eggs with a targeting vector for mouse production. The pups were genotyped by PCR followed by sequencing analysis. [file 13071_2023_5791_MOESM1_ESM.tif]

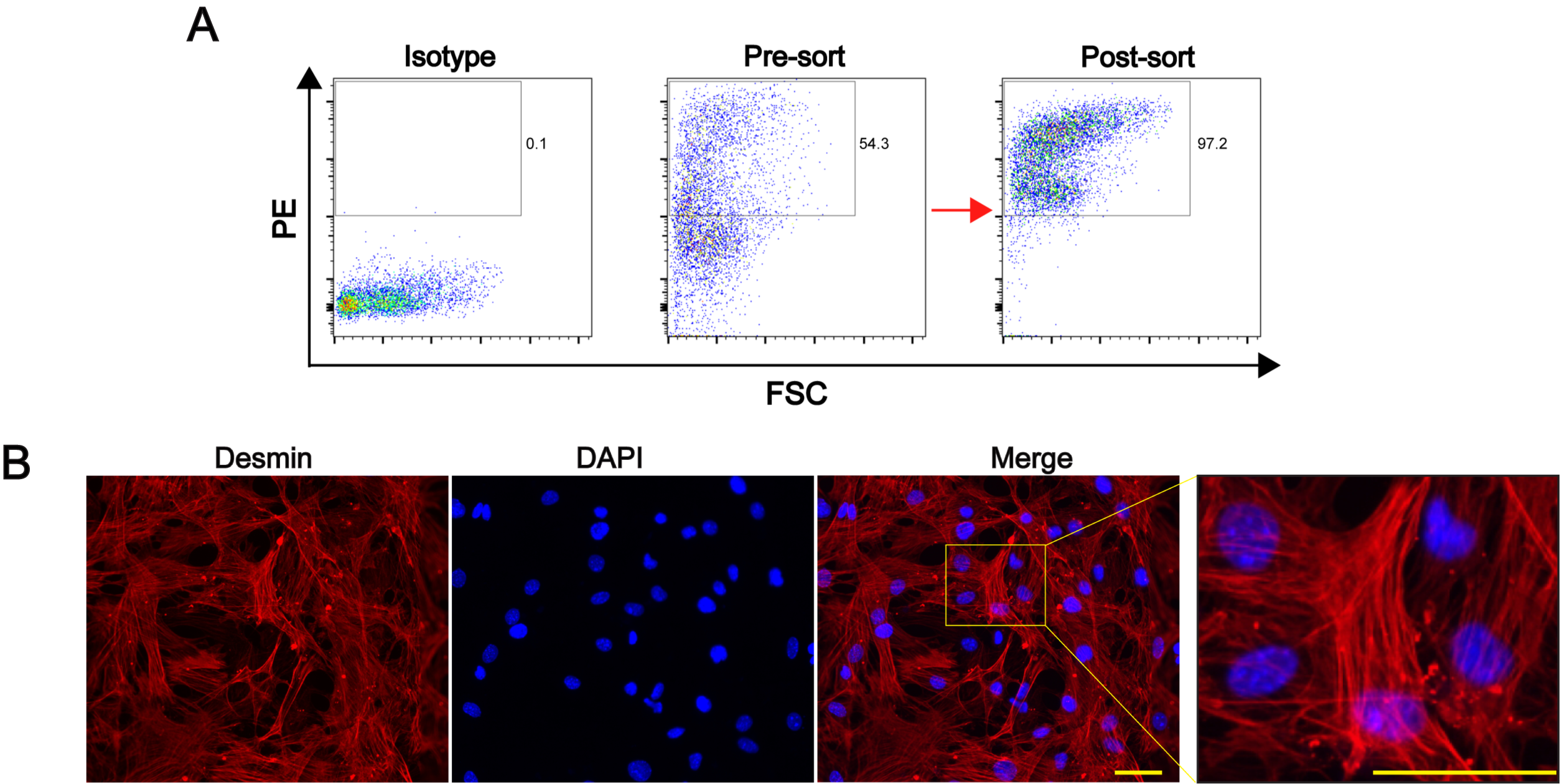

Supplement: Supplementary file 2 — Additional file 2: Figure S2. Purity of isolated HSCs. (A, B) Representative results for HSC purification by flow cytometry and immunofluorescence. Insets show a higher magnification of the outlined area. Scale bar, 50 μm. [file 13071_2023_5791_MOESM2_ESM.tif]

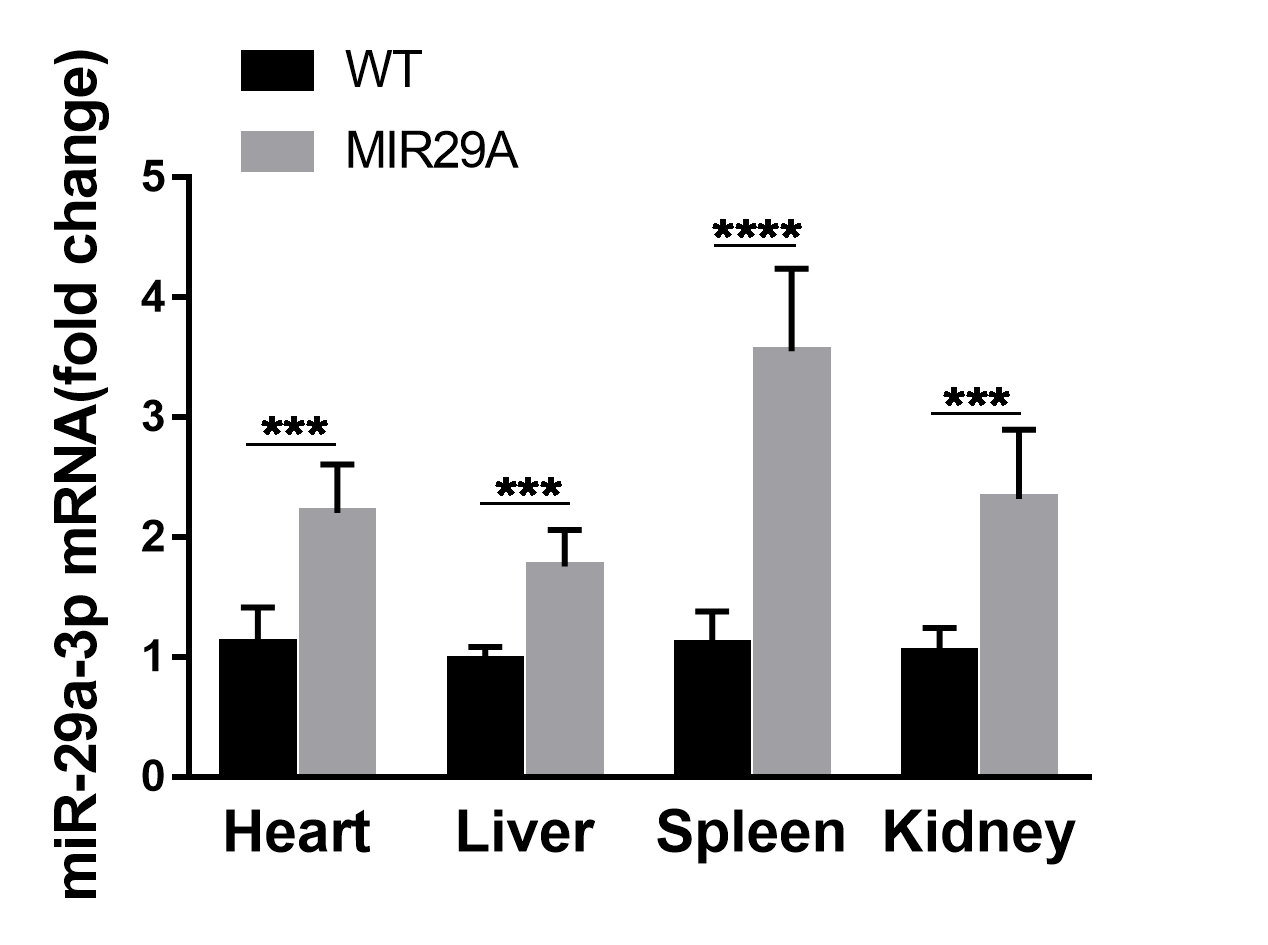

Supplement: Supplementary file 3 — Additional file 3: Figure S3. MIR29A mice had higher levels of miR-29a-3p in different organs. The miR-29a-3p expression levels in different organs of WT mice and MIR29A mice were determined by qPCR. Data are presented as the mean ± SD of three independent experiments. Significance was determined by the two-tailed Student’s t test. ***P < 0.001, ****P < 0.0001. miR-29a-3p: microRNA-29a-3p. [file 13071_2023_5791_MOESM3_ESM.tif]

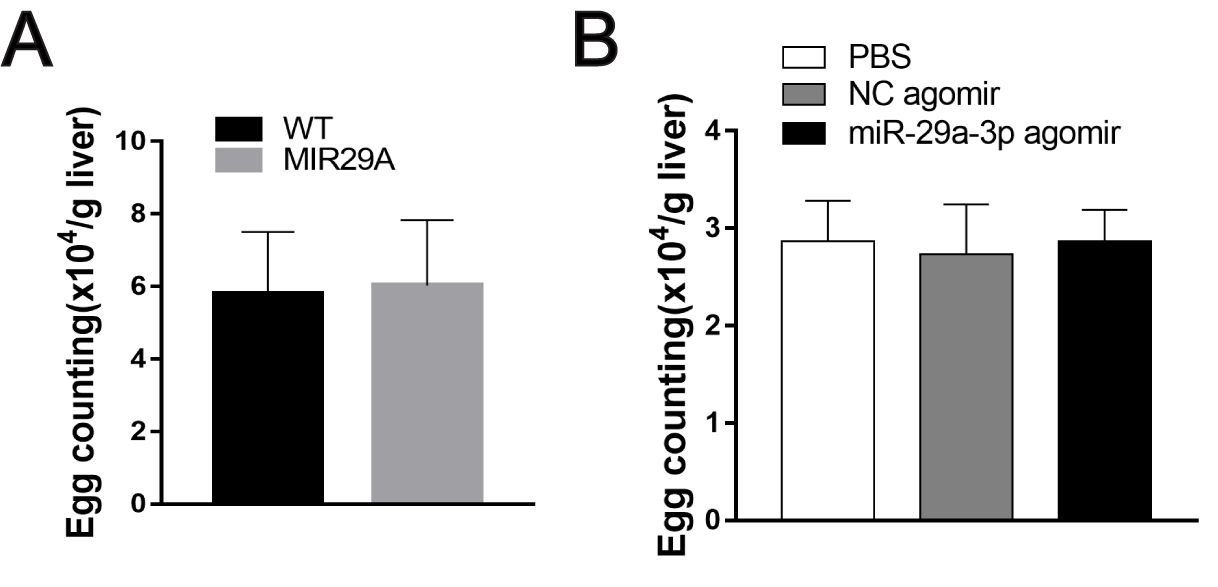

Supplement: Supplementary file 4 — Additional file 4: Figure S4. Alteration of parasite burden in the different groups during schistosome infection.Alteration of parasite burden in the infected WT mice and MIR29A mice.Alteration of parasite burden after administration of the miR-29a-3p agomir. Data are presented as the mean ± SD of three independent experiments. Significance was determined by the two-tailed Student’s t test (A) or one-way ANOVA with Tukey’s correction for comparisons between two groups (B). miR-29a-3p: microRNA-29a-3p. [file 13071_2023_5791_MOESM4_ESM.tif]
